# Two-Level Self-Thickening Mechanism of a Novel Acid Thickener with a Hydrophobic-Associated Structure during High-Temperature Acidification Processes

**DOI:** 10.3390/polym16050679

**Published:** 2024-03-02

**Authors:** Peng Li, Lei Wang, Xiaojuan Lai, Jinhao Gao, Zhiqiang Dang, Rong Wang, Fan Mao, Yemin Li, Guangliang Jia

**Affiliations:** 1 Key Laboratory of Chemical Additives for China National Light Industry, Shaanxi University of Science & Technology, Xi’an 710021, China; bs220811010@sust.edu.cn (P.L.); 4596@sust.edu.cn (J.G.); bs220811012@sust.edu.cn (Z.D.); 2 Shaanxi Agricultural Products Processing Technology Research Institute, Xi’an 710021, China; 3 National Experimental Teaching Demonstration Center of Light Chemical Engineering, Shaanxi University of Science & Technology, Xi’an 710021, China; 230112112@sust.edu.cn; 4 College of Chemistry and Chemical Engineering, Shaanxi University of Science & Technology, Xi’an 710021, China; 202009101123@sust.edu.cn; 5 Sinopec Huabei Petroleum Engineering Co., Ltd., Zhengzhou 450006, China; liyemin.oshb@sinopec.com (Y.L.); jglly@126.com (G.J.)

**Keywords:** hydrophobic association, high-temperature resistance, acid self-thickening, thickening acid, cationic polyacrylamide

## Abstract

Two acid thickeners, ADMC and ADOM, were prepared by aqueous solution polymerization using acrylamide (AM) and methacryloyloxyethyl trimethyl ammonium chloride (DMC) as raw materials, with or without the introduction of octadecyl polyoxyethylene ether methacrylate (OEMA). It was characterized by FTIR, ^1^H NMR, and the fluorescence spectra of pyrene. The double-layer thickening mechanism of ADOM was proved by comparing the thickening and rheological properties of ADMC and ADOM tested by a six-speed rotary viscometer and a HAKKE MARSIV rheometer during the acidification process. The results showed that the synthetic product was the target product; the first stage of the self-thickening ADOM fresh acid solution during high-temperature acidification was mainly affected by Ca^2+^ concentration, and the second stage of self-thickening was mainly affected by temperature. The residual viscosity of the 0.8 wt% ADOM residual acid solution was 250, 201.5, and 61.3 mPa·s, respectively, after shearing at 90, 120, and 150 °C for 60 min at a shear rate of 170 s^−1^. The thickening acid ADOM with a hydrophobic association structure has good temperature resistance and shear resistance, which can be used for high-temperature deep-well acid fracturing. In addition, no metal crosslinking agent was introduced in the system to avoid damage to its formation, and ADOM exhibited good resistance to Ca^2+^, which could provide ideas for the reinjection of the acidizing flowback fluid. It also has certain advantages for environmental protection.

## 1. Introduction

Fracturing is one of the most important means to develop unconventional resources and improve the production of oil and gas wells. However, conventional hydraulic fracturing technology has limited effects on the transformation of tight carbonate reservoirs [1]. Carbonate reservoirs have become an important field for oil and gas exploration and development. The distribution of carbonate reservoirs worldwide is also very extensive, with enormous exploration and development potential. Due to the complex geological conditions and strong heterogeneity of carbonate reservoirs, the development of carbonate reservoirs poses certain difficulties. Usually, carbonate reservoirs require reservoir transformation in order to achieve the goal of understanding the reservoir and increasing storage and production [2,3,4]. Acid fracturing is one of the important measures to transform tight carbonate reservoirs [5,6,7]. The small viscosity and large acid–rock reaction rate make the acid leak serious, and it is difficult to communicate with the formation far away from the wellbore. How to reduce acid filtration is the primary problem to improve the effect of acid fracturing [8,9].

In order to reduce acid filtration, special acid systems have been developed, including emulsified acid, variable–viscosity acid, and thickened acid. Among them, emulsified acid is the emulsification of acid liquid wrapped in oil droplets through an emulsifier. Before demulsification, the contact between the acid and rock should be avoided as much as possible so as to reduce the reaction rate of the acid rock. However, its limited application is mainly due to the high cost, large construction friction, and immature system. Variable viscosity acid is obtained by introducing a temperature-sensitive or salt-sensitive crosslinking agent into the acid solution. The crosslinking agent in variable viscosity acid begins to crosslink to increase the viscosity of the acid when the formation rises to a certain temperature (temperature sensitivity) or the acid and rock reacts to a certain extent (salt sensitivity). However, the application of variable viscosity acid is limited because the crosslinking agent currently used is mainly a metal crosslinking agent and metal ions are more harmful to the reservoir [10,11]. The viscoelastic surfactant variable viscosity acid (VES) obtained by the association between surfactants can also improve the effect of acid fracturing [12,13]. The viscoelastic surfactant variable viscosity acid (VES) obtained by the association between surfactants can also improve the effect of acid fracturing. Poor temperature resistance leads to the limited application range of the acid system. The thickened acid system has a certain viscosity and small friction, which can effectively control the acid–rock reaction rate. However, the insufficient temperature resistance of thickened acid limits its application in the acid fracturing of high-temperature wells [14,15,16,17]. After investigation, it was found that the temperature resistance of the existing thickened acid was basically below 140 °C [18,19,20,21]. In addition, because the acidizing flowback fluid contains a large amount of Ca^2+^ when it is used to prepare conventional thickened acid, the viscosity is low, the viscosity increase rate is slow or even has no viscosity, and the flowback fluid cannot be reused, resulting in greater pressure on environmental protection.

Hydrophobically associated polyacrylamide is mainly prepared by acrylamide and long-chain alkyl hydrophobic monomers. By introducing a hydrophobic side chain on the main chain, the aqueous solution can form a network structure under a hydrophobic association to improve its temperature and shear resistance [22,23,24,25,26]. Good temperature and shear resistance can meet the requirements of fracturing fluid to form cracks and joints under the action of a thermal and hydraulic transition and shorten the time of maximum crack opening displacement (COD) [27].

Herein, based on this research background, the acid thickener ADMC was prepared from acrylamide (AM) and methacryloyloxyethyl trimethyl ammonium chloride (DMC) by an aqueous solution. The acid thickener ADOM with a hydrophobic association structure was prepared by aqueous solution polymerization with AM, DMC, and octadecyl polyoxyethylene methacrylate (OEMA) as raw materials. The acid solution of ADOM can show two levels of the self-thickening phenomenon in the process of high-temperature acidification without introducing a crosslinking agent so as to avoid damaging the metal crosslinking agent during the formation. The change in Ca^2+^ concentration and temperature made hydrophobic side chains entangled with each other, which improved the viscosity and the temperature resistance of ADOM acid. It provided theoretical support for improving the temperature resistance and service temperature of thickened acid.

## 2. Materials and Methods

### 2.1. Materials

Acrylamide (AM, CP), methacryloyloxyethyl trimethyl ammonium chloride (DMC, 75%), ammonium persulfate (APS, AR), sodium bisulfite (SBS, AR), hydrochloric acid (HCl, AR), calcium chloride (CaCl_2_, AR), calcium carbonate (CaCO_3_, AR), and anhydrous ethanol (>99.7%) were purchased from Sinopharm Chemical Reagent Co., Ltd. (Shanghai, China), 2,2′-Azobis(2-methylpropionitrile) (AIBN,99%), 2,2′-Azobis(2-methylpropionamidine) dihydrochloride (V-50,97%), and pyrene (97%) were purchased from Aladdin Reagent Network (Shanghai, China) alongside octadecyl polyoxyethylene methacrylate (OEMA, 50%) were purchased from Shanghai Palimo New Materials Co., Ltd.(Shanghai, China).

### 2.2. Equipment

INVENIO Fourier transform infrared spectroscopy, AVANCE NEO 600 MHz nuclear magnetic resonance spectroscopy, the FS5 fluorescence spectrometer, six-speed rotational viscometer (ZNN-D6B), an Attension tensiometer (Sigma 702, Biolin Scientific, Espoo, Finland), HAKKE MARSIV rheometer, and a field emission scanning electron microscope were used (SEM, Regulus 8100,Tokyo, Japan).

### 2.3. Synthesis of ADMC and ADOM

**Figure 1:**
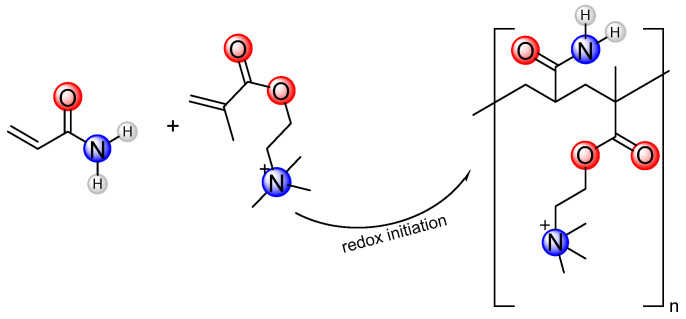
The synthetic route of the ADMC molecule.

**Figure 2:**
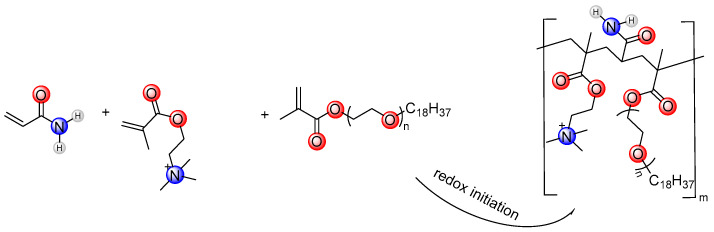
The synthetic route of the ADOM molecule.

The 213 g (3 mol) AM, 207 g (1 mol) DMC, and monomer were dissolved in 580 g of water and fully stirred. The pH of the solution was adjusted to 6 with hydrochloric acid to obtain the ADMC reaction solution. The reaction solution of ADOM was obtained by adding 3.38 g (0.01 mol) of the OEMA monomer to the above ADMC reaction solution. The system was cooled to 5 °C using an ice water bath and poured into a thermos flask. After deoxygenation by nitrogen for 30 min, 0.048 g (0.0002 mol) of ammonium persulfate, 0.022 g (0.0002 mol) of sodium bisulfite, 0.5 g of AIBN (0.003 mol), and 0.14 g (0.0005 mol) of V-50 were added, and the reaction was kept at atmospheric pressure for 4 h. After the reaction was completed, the product was granulated by a granulator, dried at 60 °C and ground to obtain the white powder products ADMC and ADOM. Purified with ethanol, vacuum drying was performed 24 h after characterization and performance testing. The synthetic routes of the self-thickening agents ADMC and ADOM are shown in Figure 1 and Figure 2, respectively.

### 2.4. Characterization and Performance Testing

#### 2.4.1. Characterization

The infrared spectra of ADMC and ADOM were measured by the INVENIO Fourier transform infrared spectrometer(Brooke (Beijing) Technology Co., Ltd.,Bei jing, China). The ^1^H NMR spectra of ADMC and ADOM in D_2_O were characterized by AVANCE NEO 600 MHz. The fluorescence intensity of pyrene in ADMC and ADOM solutions was determined by a fluorescence spectrometer. The ring method was used to test the surface tension of different concentrations of ADMC and ADOM aqueous solutions on an Attension tensiometer (Sigma 702, Biolin Scientific, Espoo, Finland).

#### 2.4.2. Rheological Properties

The apparent viscosity of 0.8% ADMC- and ADOM-thickened acids prepared with different concentrations of hydrochloric acid was tested by a six-speed rotary viscometer (ZNN-D6B, Qingdao Hengtaida Electromechanical Equipment Co., Ltd.,Qingdao, China) at 25 °C and 100 r/min, and the change rule between the apparent viscosity and acid concentration was studied. The temperature resistance, shear resistance, and viscoelasticity of the prepared sample solution were tested by the HAKKE MARSIV rheometer (Thermo Fisher Science Technology (China) Co., Ltd., Shanghai, China.)

#### 2.4.3. Measurement of Environmental Scanning Electron Microscope

The sample was a 0.8 wt% ADMC and ADOM residual acid solution prepared by vacuum sublimation freeze-drying technology. The structure of the samples was studied by field emission scanning electron microscopy (Regulus 8100,Tokyo, Japan).

## 3. Results and Discussion

### 3.1. Characterization of Polymers

#### 3.1.1. FT-IR

**Figure 3:**
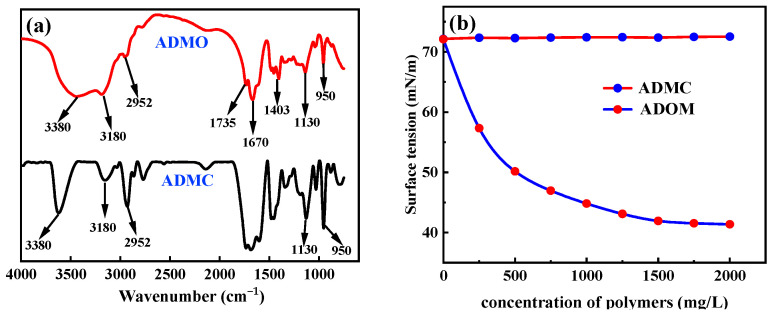

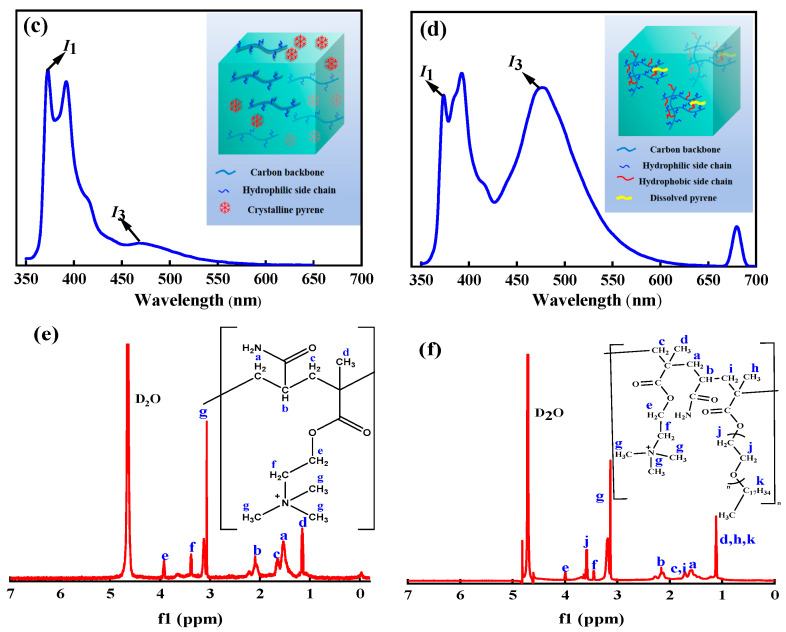
(**a**) FT-IR spectra; (**b**) surface tension of polymers; (**c**) the dispersion diagram of pyrene in ADMC aqueous solution; (**d**) the dispersion diagram of pyrene in ADOM aqueous solution; (**e**) the ^1^H NMR spectra of ADMC; and (**f**) the ^1^H NMR spectra of ADOM (In (**e**,**f**), the lowercase letters a~k represent different types of protons.).

Figure 3a shows the FT-IR spectra of ADMC and ADOM. Among them, the characteristic absorption peaks at 3350 cm^−1^, 3180 cm^−1^, and 1670 cm^−1^ are the stretching vibration peaks of N-H and C=O in AM, respectively. The characteristic absorption peaks at 2952 cm^−1^, 1735 cm^−1^, and 1130 cm^−1^ are the stretching vibration peaks of C-H, C=O, and C-O in DMC. The characteristic peak of the bending vibration of the -CH_2_-N(CH_3_)_3_ quaternary ammonium salt group was obtained in DMC. Compared with the FT-IR spectrum of ADMC, the FT-IR spectrum of ADOM has an absorption peak at 1403 cm^−1^. And the absorption peak at 1403 cm^−1^ is the bending vibration absorption peak of CH_3_ on the long chain alkyl. The above proves that ADOM was successfully synthesized.

#### 3.1.2. Surface and Interfacial Tension Tests

The side chain of the thickener ADMC for conventional thickening acid only contains hydrophilic groups such as amide groups and cationic groups. On this basis, the hydrophobic monomer OEMA was introduced to synthesize the ADOM acid thickener with a hydrophobic structure. Figure 3b is the surface tension test of ADMC and ADOM at different concentrations. ADMC only contains hydrophilic side chains, and its aqueous solution does not exhibit surface activity because its aqueous solution has a certain viscosity, and the surface tension increases. Therefore, with the increase in ADMC concentration, the surface tension of its aqueous solution is slightly larger than that of water, showing a slow upward trend. When the ADMC concentration is 2000 mg/L, the surface tension of its aqueous solution is 72.52 mN/m. The difference is that the ADOM acid thickener contains both hydrophilic side chains and hydrophobic side chains, and its aqueous solution exhibits surface activity [28]. With the increase in the ADOM concentration, the surface tension of its aqueous solution gradually decreases. When the concentration of ADOM is 2000 mg/L, the surface tension of its aqueous solution is 41.37 mN/m. The variation in the surface tension of the ADMC and ADOM aqueous solution with concentration confirmed that the acid thickener ADOM containing a hydrophobic association structure was successfully synthesized.

#### 3.1.3. Fluorescence Spectrum Test of Pyrene

In the steady-state fluorescence spectrum of pyrene, the intensity of the first peak (*I*_1_) and the intensity of the third peak (*I*_3_) are larger than those in the polar environment, which could sensitively reflect the polarity change in the microenvironment in the solution. The greater the polarity, the greater the ratio of *I*_1_/*I*_3_. Therefore, it is possible to determine whether the system contains a hydrophobic association by measuring the fluorescence intensity of pyrene. In the ADMC solution, the dispersion state of pyrene is similar to that in clear water, which is in a crystal state. As shown in Figure 3c, the ratio of *I*_1_/*I*_3_ is close to two, and there is no hydrophobic association with the microdomain. The acid thickener ADOM has a hydrophobic association structure, which can form a hydrophobic association microdomain in the water to make pyrene leave the aqueous phase and solubilize into the system. As shown in Figure 3d, the value of *I*_1_/*I*_3_ becomes smaller and close to one. The different *I*_1_/*I*_3_ values of pyrene in the ADMC and ADOM aqueous solutions prove that there are hydrophobic association microdomains in the ADOM aqueous solution, which indirectly proves that ADOM is the target product [29].

#### 3.1.4. ^1^H NMR

To further demonstrate the structure of ADMC and ADOM, the ^1^H NMR spectra of ADMC and ADOM were shown in Figure 3e and Figure 3f, respectively. From Figure 3e, the solvent peak is 4.70 ppm (D_2_O). In Figure 3e, 1.57 ppm is -CH_2_- from AM, 2.2 ppm is -CH- from AM, 1.7 ppm is CH_2_-C- from DMC, 3.3 ppm is -CH_2_-N from DMC, 3.9 ppm is O-CH_2_ from DMC, 1.2 ppm is -C-CH_3_ from DMC, and 3.1 ppm is N-(CH_3_)_3_ from DMC. In Figure 3e, the proton peak height at 1.57 ppm is twice as high as that at 1.7 ppm, while in Figure 3f, the difference between the proton peak height at 1.7 ppm and that at 1.57 ppm is smaller. This is because part of the proton peak comes from -CH_2_-C- in OEMA. In Figure 3e, the height of the proton peak at 1.2 ppm is twice as high as that at 1.7 ppm, but in Figure 3f, the ratio of the height of the proton peak at 1.2 ppm to the height of the proton peak at 1.7 ppm is significantly larger. This is because some of the proton peaks are provided by-C-CH_3_ and (-CH_2_-)_17_ from OEMA; 3.5 ppm is-OCH_2_CH_2_-O from OEMA. The results show that the synthesized product was consistent with the target product. The ^1^H NMR spectrum of ADMC and ADOM data is further summarized in Table 1.

### 3.2. Study on Thickening Mechanism of ADMC and ADOM Acid Solution

The external factors affecting the rheological properties of hydrophobic-associated polymers are mainly salt and temperature.

#### 3.2.1. Effect of Salt

During the acid–rock reaction, the concentration of H^+^ decreases, and the concentration of Ca^2+^ increases. And, in general, divalent ions have a greater effect on the rheological properties of polymer solutions than monovalent ions. It is necessary to study the effect of salt on shear behavior. Salt increases the polarity of the solution and enhances the hydrophobic association, which means that as the network structure increases, the viscosity of the solution increases, and the temperature and shear resistance is enhanced [30,31]. The viscosity of the fresh acid solution and residual acid solution of ADMC, ADOM, and the viscosity of simulated residual acid solution of ADOM conform to this change rule.

The 0.8 wt% ADMC fresh acid solution was obtained by completely dissolving 1.6 g of ADMC in 198.4 g of 20 wt% hydrochloric acid. The residual acid solution with a hydrochloric acid mass fraction of 15%, 10%, 5%, and ~0% was obtained by completely swelling the 0.8% ADMC fresh acid solution and adding 13.7, 27.4, 41.1, and 54.8 g of CaCO_3_ powder according to the reaction relationship between hydrochloric acid and calcium carbonate (molar ratio of 2:1). The fresh acid solution of ADOM and the residual acid solution with different reaction degrees were obtained by the same steps. The simulated residual acid solution was prepared with CaCl_2_ and different concentrations of hydrochloric acid according to the reaction relationship between hydrochloric acid and calcium carbonate (molar ratio of 2:1). For example, the 10% residual acid solution was obtained by consuming 10 g (0.274 moL) of HCl and generating 15.07 g (0.137 moL) of CaCl_2_ from 100 g of fresh acid (20 wt%) during the acid–rock reaction. Therefore, the simulated 10% residual acid solution was prepared by 84.93 g of 10% hydrochloric acid and 15.07 g of CaCl_2_. The ratio of hydrochloric acid and CaCl_2_ in the residual acid solution with different acid–rock reaction degrees is shown in Table 2.

**Figure 4:**
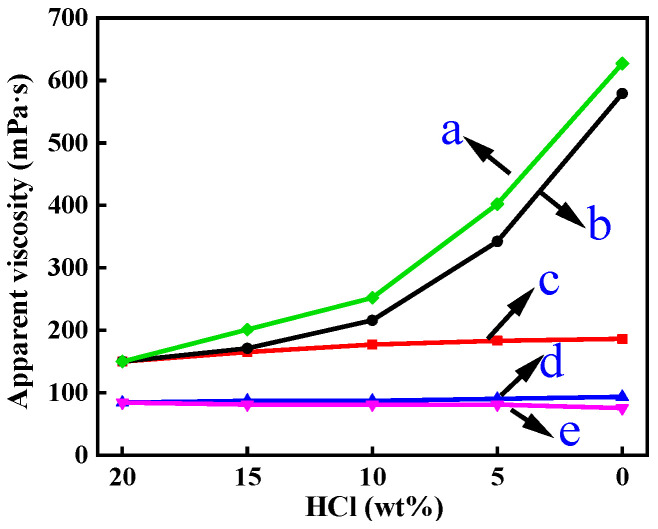
Viscosity change in the ADMC, ADOM acid solution, and simulated residual acid solution of ADOM: (a) simulated 0.8% ADOM residual acid solution; (b) 0.8% ADOM residual acid solution; (c) 0.8% ADOM acid solution; (d) 0.8% ADMC acid solution; and (e) 0.8% ADMC residual acid solution.

In Figure 4, the viscosity change rule of b (150~579 mPa·s) and e (84~75 mPa·s) is opposite. The reason for this situation is that there is an increase in the Ca^2+^ concentration in the acid–rock reaction, the polarity of the acid solution is enhanced, and the hydrophobic chain segments are entangled with each other. In addition, c (150~190 mPa·s) and d (84~93 mPa·s) have the same change rule. This is because only the concentration of H^+^ is reduced, Ca^2+^ is not introduced, and free H^+^ affects the thickening ability of the acrylamide group in water. This is also the reason why the end viscosity of a (627 mPa·s) is greater than that of b (579 mPa·s). By comparing the viscosity variation in the residual acid solution, it is proved that Ca^2+^ is the main factor affecting the viscosity of the ADOM self-thickening acid solution.

**Figure 5:**
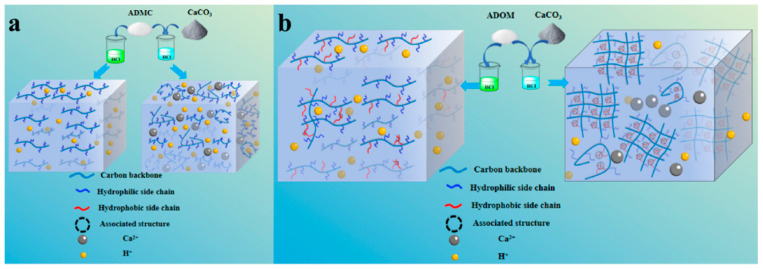
The schematic diagram of the aggregation state of the molecular chains of ADMC and ADOM during the acid–rock reaction: (**a**) ADMC; (**b**) ADOM.

Figure 5a,b is the diagram of the change in the molecular chain aggregation state of the acid solution of ADMC and ADOM in the process of the acid–rock reaction. In Figure 5a, the molecular chain is in a normal swelling state in the ADMC fresh acid solution. During the acid–rock reaction, with the increase in Ca^2+^ concentration in the solution, Ca^2+^ makes the polarity of the solution stronger, interacts with the cationic side chain (from DMC) in the ADMC molecular chain, and makes the ADMC molecular chain curl. The macroscopic performance is that the viscosity of the acid solution decreases. The swelling state of the ADOM molecular chain in a fresh acid solution is shown in Figure 5b as normal swelling, which is similar to the swelling state of ADMC in a fresh acid solution. However, Figure 5a,b also show the differences in the microstructures between ADOM and ADMC. On the one hand, during the acid–rock reaction, Ca^2+^ makes the polarity of the solution stronger, and the presence of cationic groups makes the molecular chains curl. On the other hand, different from ADMC, there are both hydrophilic side chains and hydrophobic side chains (as shown by the red chains in Figure 5b) in the ADOM polymer chains as acid thickeners. After the complete reaction between CaCO_3_ and hydrochloric acid, there are both intramolecular and intermolecular hydrophobic associations in the ADOM molecular chain aggregates, forming a microscopic network structure (as shown by the right half of Figure 5b) that leads to an increase in solution viscosity; at the same time, the hydrophobic chain segments in the ADOM acid solution are entangled and associated with each other due to the increase in polarity. The macroscopic performance is that the viscosity of the acid solution increases, and the temperature resistance is enhanced.

**Figure 6:**
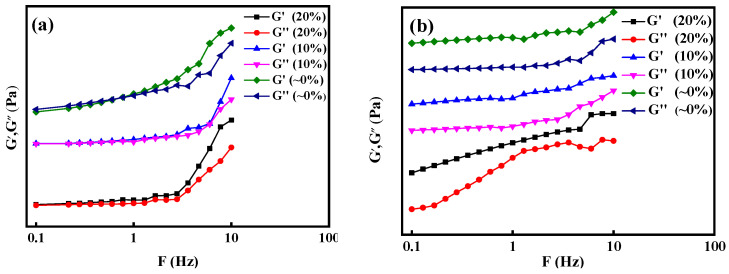
Viscoelasticity of ADMC and ADOM acid solution. (**a**) ADMC; (**b**) ADOM.

Figure 6 shows the viscoelastic tests of 20% fresh acid solution, 10% residual acid solution, and ~0 residual acid solution of ADMC and ADOM, respectively. In Figure 6a, with the decrease in acid concentration, the increase in Ca^2+^ concentration enhanced the polarity of the solution, interacted with the cationic group in DMC, curled the ADMC molecular chain, reduced the difference between G′ and G″ of the solution, and weakened the elasticity of the solution. Among them, the viscoelastic test of the ~0% residual acid solution of ADMC shows that G″ is greater than G′ in the frequency range of 0.1~1 Hz, indicating that the solution is in a viscous fluid state at this frequency. As shown in Figure 6b, the viscoelasticity of ADOM acid solutions with different acid concentrations was compared and tested. With the decrease in acid concentration, the increase in Ca^2+^ concentration in the solution enhanced the polarity of the solution, forcing the hydrophobic segments to intertwine and associate with each other. Under the action of shear stress, the association point continuously breaks and re-associates. Therefore, the G´ of ADOM acid solution increases with the decrease in acid concentration, and the overall performance is more elastic.

#### 3.2.2. Effect of Temperature

For conventional water-soluble polymers, the viscosity of the solution decreases with increasing temperature and obeys the Arrhenius formula [32], as shown in Formula 1. However, the hydrophobic association is an entropy-driven process. The increase in temperature is beneficial to the association and the formation of a network structure and the improvement of the temperature resistance and shear resistance of the solution [33,34,35].

**Figure 7:**
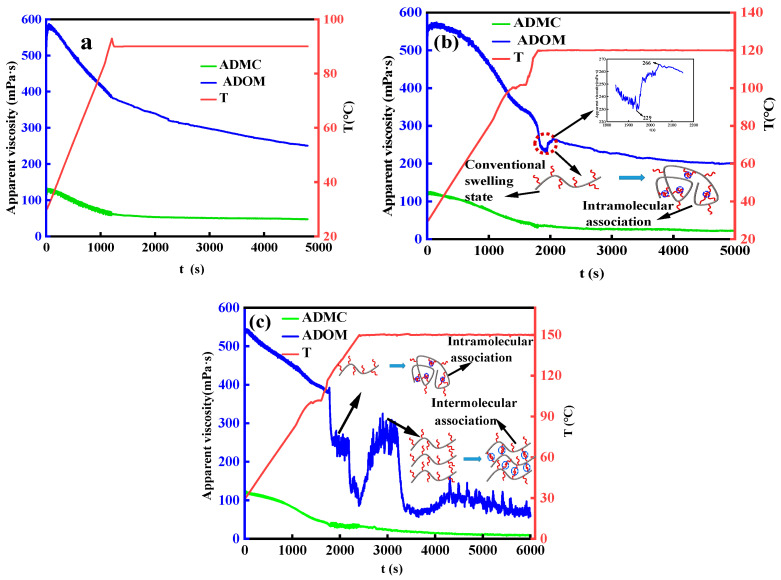
Temperature and shear resistance tests of the 0.8% ADMC and ADOM residual acid solution. (**a**) 90 °C; (**b**) 120 °C; and (**c**) 150 °C.

Figure 7 shows the rheological properties of ADMC and ADOM residual acid solutions with ~0%, including the viscosity changes in the solution at 90 °C, 120 °C, and 150 °C at a shear rate of 170 s^−1^. It can be seen from the green curve in Figure 7 that the viscosity of the ADMC residual acid solution continues to decrease with the increase in shear temperature and shear time. The residual viscosities after shearing for 60 min at three temperatures were 47, 20, and 9 mPa·s, respectively.

In addition, the blue curve in Figure 7 is the shear performance test curve of ADOM residual acid solution at three temperatures. It can be seen from Figure 7a that when the ADOM residual acid solution was subjected to a shear performance test at 90 °C, the viscosity of the residual acid solution continued to decrease with the increase in temperature and the extension of shear time. The residual viscosity of the residual acid solution was 250 mPa·s after shearing for 60 min. It can be seen from the blue curve in Figure 7b that when the temperature rose to 120 °C, the viscosity of the residual acid solution of ADOM increased from 239 mPa·s to 266 mPa·s. This phenomenon was due to the association effect in the molecular chain of the residual acid solution when the temperature was raised to 120 °C, and the macroscopic performance is that the viscosity of the solution increased. The residual viscosity of the residual acid solution was 201.5 mPa·s after shearing at 120 °C for 60 min. The shear of the ADOM residual acid solution at 150 °C is shown in the blue curve in Figure 7c. It can be seen from this figure that when the temperature rises to 120 °C, the viscosity change rule is the same as the blue curve in Figure 7b, and the viscosity of the solution increases slightly. In addition, when the temperature rose to 150 °C, the viscosity of the solution increased from 85 mPa·s to 325 mPa·s, which is greater than the increase in the viscosity of the acid solution when the temperature rose to 120 °C. The viscosity of the thickened acid solution increased because, at this temperature, the long-chain alkyl groups were entangled with each other between the molecular main chains, and this was stronger than the association effect within the molecular chain. Moreover, the spatial network structure formed by entanglement was better than the temperature resistance of simple long-chain macromolecules [36]. With the extension of shear time, the spatial network structure was gradually destroyed, and the viscosity of the solution decreased. After shearing at 150 °C for 60 min, the residual viscosity was 61.3 mPa·s.

**Figure 8:**
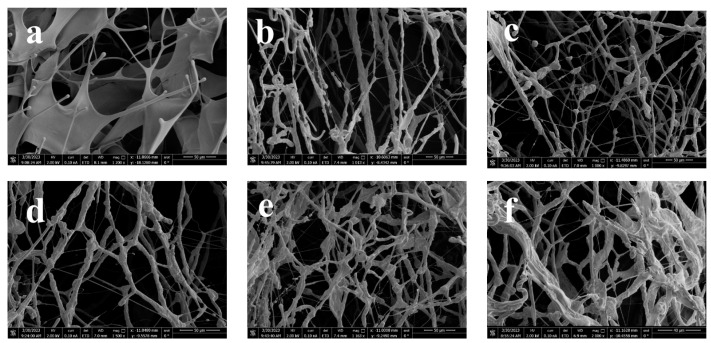
**An** 0.8 wt% ADOM-simulated residual acid solution scanning electron microscope; (**a**) 20% fresh acid solution; (**b**) 15% residual acid solution; (**c**) 10% residual acid solution; (**d**) 5% residual acid solution; (**e**) ~0% residual acid solution; (**f**) and the ~0% residual acid solution of ADOM was magnified 2000 times.

In Figure 8a–f, the field emission scanning electron microscope image of the ADOM molecular chain in the process of a simulated acid salt reaction is shown. Among them, Figure 8a shows the 20% fresh acid solution of ADOM. In this figure, the polymer molecules are flakes with many-branched chains but few association points. The field emission scanning electron microscope images of residual acid solutions with hydrochloric acid concentrations of 15%, 10%, 5%, and ~0% are shown in Figure 8b–f. As the concentration of hydrochloric acid decreases, the concentration of Ca^2+^ in the solution increases, and the polarity of the solution increases. Ca^2+^ is adsorbed into the polymer molecular chain, forcing the hydrophobic segments to intertwine and associate with each other, and thus, the network structure and association points gradually increase. The macroscopic performance is the increase in solution viscosity, which is the same as the change law of apparent viscosity in Figure 4 and Figure 5.

#### 3.2.3. Formula of rheology behavior

(1) η=Aexp(EηRT)

where **η** is the viscosity (mPa·s), ***A*** is a constant, ***E_η_*** is the activation energy of the viscous flow (J·mol^−1^), ***R*** is the gas constant (J/(mol*K)), and ***T*** is the thermodynamic temperature (K).

## 4. Conclusions

In conclusion, a novel acid thickener ADOM with a hydrophobic association structure was prepared by aqueous solution polymerization using AM, DMC, and OEMA as raw materials. The viscosity of the 0.8 wt% ADOM acid thickener in 20 wt% hydrochloric acid is 150 mPa·s. At the same time, the mechanism of the two-level self-thickening phenomenon of the ADOM fresh acid solution during high-temperature acidification was studied. Specifically, a thickening phenomenon occurs when the H^+^ in the acid solution is consumed and the Ca^2+^ concentration increases during the acid–rock reaction. Another thickening phenomenon is that the temperature changes the aggregation state of the ADOM molecular chain in the residual acid solution to increase its viscosity, which occurs in the process of acid migration away from the wellbore. This conclusion was obtained by the phenomenon that the viscosity of the ADOM residual acid solution increases at 120 and 150 °C in the temperature and shear resistance test. The mechanism of the thickening phenomenon of these two levels is proved in this paper. The residual viscosity of 0.8% ADOM residual acid solution was 250, 201.5, and 61.3 mPa·s, respectively, after shearing at 90, 120, and 150 °C for 60 min at the shear rate of 170 s^−1^. In addition, because the liquid, after acidizing flowback, contains a large amount of Ca^2+^, the viscosity is small when it is used to prepare conventional thickening acid, which affects the construction effect. Ca^2+^ has a good thickening ability for the thickening acid of ADOM and provides technical support for the reinjection of acidizing flowback fluid, which can reasonably develop and utilize water resources. In general, the hydrophobic associative acid thickener ADOM, with certain environmental protection effects and good temperature resistance, has a good application prospect in high-temperature acidification construction.

## Figures and Tables

**Table 1:** The ^1^H NMR spectrum of ADMC and ADOM data.

δ, ppm	Protons from ADMC	Protons from ADOM
1.2	d, -C-CH_3_, (DMC)	d, h, k, -C-CH_3_, (DMC), -C-CH_3_, (-CH_2_-)_17_, (OEMA)
1.5	a, -CH_2_-, (AM)	a, -CH_2_-, (AM)
1.7	c, -CH_2_-C-, (DMC)	c, i, -CH_2_-C-, (DMC), -CH_2_-C-, (OEMA)
2.2	b, -CH-, (AM)	b, -CH-, (AM)
3.1	g, N-(CH_3_)_3_, (DMC)	g, N-(CH_3_)_3_, (DMC)
3.3	f, -CH_2_-N-, (DMC)	f, -CH_2_-N-, (DMC)
3.9	e, O-CH_2_-, (DMC)	e, O-CH_2_-, (DMC)
3.5	/	j, (-CH_2_CH_2_-O-)_n_, (OEMA)

**Table 2:** Configuration of residual acid solution with different acid–rock reaction degrees.

Simulated Concentration of Hydrochloric Acid	20%	15%	10%	5%	~0%
Addition of hydrochloric acid	100 g of 20% hydrochloric acid	92.47 g of 15% hydrochloric acid	84.94 g of 10% hydrochloric acid	77.41 g of 5% hydrochloric acid	69.88 g of water
Addition of CaCl_2_	0 g	7.53 g	15.06 g	22.59 g	30.12 g

Note: The percentages in the table are mass fractions.

## Data Availability

The data that support the findings of this study are available from the corresponding author, [P.L.], upon reasonable request.

## References

[1] Islamov S.R., Bondarenko A.V., Gabibov A.F., Mardashov D.V. (2021). Polymer compositions for well killing operation in fractured reservoirs. Advances in Raw Material Industries for Sustainable Development Goals.

[2] Zhang Y., Chen A., Mao J.C., Qin S.-H., Li J., Yang X.-J., Lin C., Huang Z.-Y., Liu Y.-F. (2023). Preparation of a functional fracturing fluid with temperature-and salt-resistance, and low damage using a double crosslinking network. Pet. Sci..

[3] Islamov S.R., Bondarenko A.V., Mardashov D.V. (2019). Substantiation of a well killing technology for fractured carbonate reservoirs. Proceedings of the Youth Technical Sessions Proceedings: VI Youth Forum of the World Petroleum Council–Future Leaders Forum.

[4] Guo B., Liu X., Tan X. (2017). Petroleum Production Engineering.

[5] Zhao G., Dai C.L., Chen A., Yan Z., Zhao M. (2015). Experimental study and application of gels formed by nonionic polyacrylamide and phenolic resin for in-depth profile control. J. Pet. Sci. Eng..

[6] Jain R., Mahto V. (2015). Evaluation of polyacrylamide/clay composite as a potential drilling fluid additive in inhibitive water based drilling fluid system. J. Pet. Sci. Eng..

[7] Zhou L., Zou C.J., Gu T., Li X., Shi Y. (2017). Cucurbit[7]uril-modified intelligent polymer as acid thickening agent for unconventional reservoir recovery. J. Pet. Sci. Eng..

[8] Choi J., Kim J., Kim K., Yang S.-T., Kima J.-I., Jon S. (2007). A rationally designed macrocyclic cavitand that kills bacteria with high efficacy and good selectivity. Chem. Commun..

[9] Guo B., Li Z., Guo J.C., Zhang R., Zhou C., Wu L., Ye J., Zeng J. (2021). Effect of different types of stimulation fluids on fracture propagation behavior in naturally fractured carbonate rock through CT scan. J. Pet. Sci. Eng..

[10] Lynn J.D., Nasr-El-Din H.A. A Core Based Comparison of The Reaction Characteristics of Emulsified And In-Situ Gelled Acids In Low Permeability, High Temperature, Gas Bearing Carbonates. Proceedings of the SPE International Symposium on Oilfield Chemistry.

[11] Nasr-El-Din H.A., Al-Mohammad A.M., Al-Shurei A.A., Merwat N.K., Erbil M.M., Samuel M. (2006). Restoring the injectivity of water disposal wells using a viscoelastic surfactant-based acid. J. Pet. Sci. Eng..

[12] Neto A., Silva C., Torres R.S., Farias R.L., Prata F.G., Souza L.A., Pereira A.Z., Calderon A., Sandes E.F. Self-Diverting Acid for Effective Carbonate Stimulation Offshore Brazil: A Successful History. Proceedings of the SPE European Formation Damage Conference & Exhibition.

[13] Li H., Shi Y. (2022). Synthesis and performance of temperature-and acid-resistant ternary-copolymer thickener. Mater. Chem. Phys..

[14] Guo B., Zeng M.Y., Wang K.J., Li X., Guo J. (2021). Effect of fiber on the rheological properties of gelled acid. J. Pet. Explor. Prod. Technol..

[15] Zhao G., Dai C., Li W., Yan Z., Zhao M. (2016). Research on a temporary plugging agent based on polymer gel for reservoir acidification. J. Pet. Explor. Prod. Technol..

[16] Qiao M.J., Cao G.S., Gao Y.J., Ge L. (2014). The Research and Application of Injection Wells Acidification with Temporary Plugging Agent. Appl. Mech. Mater..

[17] Tian H., Quan H., Huang Z., Duan W., Deng S. (2019). Polymeric and non-crosslinked acid self-thickening agent based on hydrophobically associating water-soluble polymer during the acid rock reaction. J. Appl. Polym. Sci..

[18] Quan H., Zhen X., Wu Y., Duan W. (2022). Effect of non-crosslinked polymer (ADDA) on acid-rock reactions: Synthesis and thickening laws. J. Polym. Res..

[19] Ge Y.R., Zhao Z.C., Cheng X.L., Chen T., Liu T., Guo X. (2021). Research of a novel double cross-linking fracturing fluid. J. Pet. Explor. Prod. Technol..

[20] Tang W.Y., Zou C.J., Peng H., Wang Y., Shi L. (2021). Influence of Nanoparticles and Surfactants on Stability and Rheological Behavior of Polymeric Nanofluids and the Potential Applications in Fracturing Fluids. Energy Fuels.

[21] Zhao J.Z., Yang B., Mao J.C., Zhang Y., Yang X., Zhang Z., Shao Y. (2018). A Novel Hydrophobic Associative Polymer by RAFT-MADIX Copolymerization for Fracturing Fluids with High Thermal Stability. Energy Fuels.

[22] Wu R., Zhang S., Chen Y., Chen H., Wang M., Tan Y. (2022). Salt endurable and shear resistant polymer systems based on dynamically reversible acyl hydrazone bond. J. Mol. Liq..

[23] Wang Z., Cui H., Liu M., Grage S.L., Hoffmann M., Sedghamiz E., Wenzel W., Levkin P.A. (2022). Tough, transparent, 3D-printable, and self-healing poly (ethylene glycol)-gel (PEGgel). Adv. Mater..

[24] Patel V., Trivedi J., Sharma T. (2023). Influence of hydrophobic association in the aqueous media on the rheology and polymer conformation of associative polymers. Polym. Bull..

[25] Yang E. (2021). The Synthesis of Associative Copolymers with Both Amphoteric and Hydrophobic Groups and the Effect of the Degree of Association on the Instability of Emulsions. Polymers.

[26] Feng Y., Billon L., Grassl B., Khoukh A., François J. (2002). Hydrophobically associating polyacrylamides and their partially hydrolyzed derivatives prepared by post-modification. 1. Synthesis and characterization. Polymers.

[27] Abdollahipour A., Marji M.F. (2020). A thermo-hydromechanical displacement discontinuity method to model fractures in high-pressure, high-temperature environments. Renew. Energy.

[28] Fan M., Wang L., Li J., He P., Lai X., Gao J., Liu G., Wen X. (2022). Preparation of supramolecular viscoelastic polymers with shear, temperature, and salt resistance/sensitivity by amphiphilic functional monomer modification. Polym. Test..

[29] Feng Y.J., Billon L., Grassl B., Bastiat G., Borisov O., François J. (2005). Hydrophobically associating polyacrylamides and their partially hydrolyzed derivatives prepared by post-modification. 2. Properties of non-hydrolyzed polymers in pure water and brine. Polymer.

[30] Selb J., Biggs S., Renoux D., Candau F. (1996). Hydrophobic and Electrostatic Interactions in Water-Soluble Associating Copolymers.

[31] Ma X., Huang Q., Zhou Z., Mu Y. (2022). Synthesis and evaluation of water-soluble fracturing fluid thickener based on hydrophobic association. Mater. Lett..

[32] Seymour R.B., Carraher C.E. (1984). Structure—Property Relationships in Polymers.

[33] Amis E.J., Hu N., Seery T.A.P., Hogen-Esch T.E., Yassini M., Hwang F. (1996). Associating Polymers Containing Fluorocarbon Hydrophobic Units.

[34] Taylor K.C., Nasr-EI-Din H.A. (1995). Water-soluble hydrophobically associating polymers for improved oil recovery: A literature review. Proceedings of the SPE International Conference on Oilfield Chemistry.

[35] Uhl J.T., Ching T.Y., Bae J.H. (1995). A Laboratory Study of New, Surfactant-Containing Polymers for High Salinity Reservoirs. SPE Adv. Technol. Ser..

[36] Salami O.T., Plank J. (2012). Synthesis, effectiveness, and working mechanism of humic acid-sodium 2-acrylamido-2-methylpropane sulfonate-co-N,N-dimethyl acrylamide-co-acrylic acid graft copolymer as high-temperature fluid loss additive in oil well cementing. J. Appl. Polym. Sci..

